# Early development of the root-knot nematode *Meloidogyne incognita*

**DOI:** 10.1186/s12861-016-0109-x

**Published:** 2016-04-28

**Authors:** Alejandro Calderón-Urrea, Bartel Vanholme, Sandra Vangestel, Saben M. Kane, Abdellatif Bahaji, Khavong Pha, Miguel Garcia, Alyssa Snider, Godelieve Gheysen

**Affiliations:** Department of Biology, College of Science and Mathematics, California State University, 2555 East San Ramon Avenue, Fresno, CA 93740 USA; Faculty of Bioscience Engineering, Department of Molecular Biotechnology, BW14, Ghent University, Coupure links 653, B-9000 Ghent, Belgium; Department of Plant Systems Biology, VIB, Technologiepark 927, B-9052 Ghent, Belgium; Faculty of Sciences, Department of Biology, Ghent University, K.L. Ledeganckstraat 35, B-9000 Ghent, Belgium; Instituto de Agrobiotecnologia (CSIC/UPNA/Gobierno de Navarra), Ctra. de mutilva baja, s/n 31192, Mutilva Baja, Navarra Spain; Department of Plant Biotechnology and Bioinformatics, Ghent University, Technologiepark 927, B-9052 Ghent, Belgium; Biochemistry, Molecular, Cell, and Developmental Biology Graduate Group, Department of Microbiology and Molecular Genetics, University of California, 1 Shields Avenue, Davis, CA 95616 USA; Department of Biology, James H. Clark Center, Stanford University, 318 Campus Drive, W200, Stanford, CA 94305 USA; IVIGEN Los Angeles, 406 Amapola Ave. Suite 215, Torrance, CA 90501 USA

**Keywords:** Cell lineage, early development, embryogenesis, 4D-microscopy, Nematoda

## Abstract

**Background:**

Detailed descriptions of the early development of parasitic nematodes are seldom available. The embryonic development of the plant-parasitic nematode *Meloidogyne incognita* was studied, focusing on the early events.

**Results:**

A fixed pattern of repeated cell cleavages was observed, resulting in the appearance of the six founder cells 3 days after the first cell division. Gastrulation, characterized by the translocation of cells from the ventral side to the center of the embryo, was seen 1 day later. Approximately 10 days after the first cell division a rapidly elongating two-fold stage was reached. The fully developed second stage juvenile hatched approximately 21 days after the first cell division.

**Conclusions:**

When compared to the development of the free-living nematode *Caenorhabditis elegans*, the development of *M. incognita* occurs approximately 35 times more slowly. Furthermore, *M. incognita* differs from *C. elegans* in the order of cell divisions, and the early cleavage patterns of the germ line cells. However, cytoplasmic ruffling and nuclear migration prior to the first cell division as well as the localization of microtubules are similar between *C. elegans* and *M. incognita*.

**Electronic supplementary material:**

The online version of this article (doi:10.1186/s12861-016-0109-x) contains supplementary material, which is available to authorized users.

## Background

The root-knot nematode *Meloidogyne incognita* (Tylenchida) is an economically important plant parasite with a wide host range, and abundant field populations can develop quickly under appropriate conditions. This rapid population growth is mainly due to the completion of several generations during a single growing season, combined with the high females fecundity. The exact number of eggs produced varies depending on environmental conditions. Under favorable conditions, a single female may produce 500–2000 eggs [1]. The eggs have transparent protective chitin-containing shells and are deposited by the female in a desiccation resistant gelatinous matrix secreted by the female. Although males do exist, reproduction occurs exclusively via mitotic parthenogenesis (apomixis) [2].

Since there is no sperm contribution during reproduction in *M. incognita*, questions concerning determination of axis polarity and early events during development are very important, particularly considering the fact that these events are significant during the evolution of developmental patterns [3, 4]. In *C. elegans*, upon fertilization, the egg continues through meiosis, eventually leading to formation of the zygote and the first zygotic cellular division. The position of sperm entry into the oocyte designates the future posterior region of the developing embryo [5]. The sperm also brings the centrosome needed for cell division, and paternal pronucleus that are required to carry out the formation of the zygote [6]. The centrosome is required for microtubule formation, and is also needed to position the pronuclei for fusion. After the sperm enters, a series of events occurs that organize the cell. Among these are cytoplasmic ruffling and nuclear migration. In *C. elegans* cytoplasmic ruffling occurs after the moment of fertilization. This process involves movement of cytoplasmic material from the posterior side of the egg to the anterior region, or vice versa [7]. Inside the *C. elegans* early embryo, which at this point is called a P_o_ cell, there are a series of movements referred to as cortical flows, which appear physically as pseudocleavages and invaginations in the cell [7]. Cortical flow is a result of contractions of the cytoskeleton, which move PAR proteins, such as PAR-3, in the anterior direction, establishing cell polarity [8]. PAR-3 begin to locate to the anterior region [9, 10], while PAR-2 and P-granules move towards the posterior region, which was defined as such when the sperm entered the egg in that region [11]. PAR-3 and PAR-2 proteins thus define the boundary of the anterior and posterior region of the single-celled embryo [12].

One of the major differences between *C. elegans* and *M. incognita* is the role of the sperm. Although sperm is not required for initiation of embryogenesis in *C. elegans*, a sexually reproducing nematode, the sperm does provide the centrosome, which is required for the first cell division. Furthermore, sperm entry is associated with proper positioning, determining anteriorposterior (A-P) polarity and leading to the future asymmetric cell division. It is not currently known what triggers development in the eggs off *M. incognita*. To better understand *M. incognita*’s reproductive mechanism, several cytological events during gametogenesis and oogenesis have been studied in detail (see [13]). In a SEM study, distinct developmental stages of embryo development could be distinguished [14]. Another study followed the early stages of development of *M. incognita*, along with 36 other species of Nematoda, in order to provide a survey of early development for phylogenetic purposes; this early work reports that *M. incognita* has a synchronous pattern of development (i. e. the four blastomeres present are the same generation), that the first four blastomeres have the same size, and that they organize in tandem [15]. However, there are no previous studies that investigated early cell lineages, including the timing of specific developmental events. This is mainly due to both the within-gall inaccessibility of this obligatory parasite and its slow development, making observations cumbersome and time consuming. In this study we documented the early developmental events of *M. incognita*, using 4D-microscopy, to draw comparisons with the well-characterized development of *C. elegans.*

## Methods

### Culturing nematodes and collecting eggs

Susceptible tomato plants (Rutger’s Select, Tomatoes, Augusta, GA) were grown in 0.5 l autoclaved white sand amended with slow release fertilizer (Osmocote® 19-6-12 formula, The Scotts Company LLC). The plants were grown at 28 °C with a 16 h light/8 h dark photoperiod and watered daily. Approximately 1-week-old tomato plants (10 cm shoot and approximately 4–5 cm long roots) were infected with pre-parasitic juveniles of *M. incognita* (race 1). The roots of an infected tomato plant (8–10 weeks post infection) were washed free of soil and heavily galled roots were gently chopped in M9 buffer (90 mM Na_2_HPO_4_, 22 mM KH_2_PO_4_, 9 mM NaCl and 19 mM NH_4_Cl) to release the eggs, shaken vigorously for 5 min with 10 % bleach, and subsequently poured through a 250 μm mesh screen. Eggs were collected from the flow-through on a 25 μm mesh screen and further purified by centrifugation for 10 min on a 35 % sucrose gradient at 500 × g. The egg-containing fraction was then subjected to two 10 min treatments in 10 % bleach followed by centrifugation at 500 × g for 5 min and several rinses in sterile distilled (DI) water.

### Slide preparation

Eggs from one infected tomato plant were harvested as described, observed with an inverted compound microscope and isolated using a drawn-out Pasteur pipette. The selected eggs were transferred to a microscope slide carrying a thin 5 % agar pad. The eggs were covered with a coverslip and sealed with petroleum jelly.

### DAPI staining

Approximately 10^5^ fresh embryos were fixed in Histochoice Tissue Fixative MB (Amresco, Solon, OH) for 2 h and cleared in Histochoice Clearing Agent (Amresco, Solon, OH). 4',6-Diamidino-2-phenylindole (DAPI) was added to a final concentration of 0.1 μg/ml. The stained eggs were transferred to a slide, covered with a coverslip and sealed with clear nail polish. Eggs were viewed with an Olympus IX70 inverted microscope using a 40× (NA 0.75) objective lens and settings for both Nomarski (Differential Interference Contrast) and fluorescent DAPI imaging. Squash preparations to count nuclei of developing embryos were prepared as reported previously [16].

### 4D-microscopy

Developing embryos (*n* = 242) were observed at room temperature (22+/-1 °C) by Nomarski (Differential Interference Contrast microscopy) or bright field optics using a Nikon inverted microscope with a 40× oil objective (NA 1.3). A motorized stage controller and automatic shutter were incorporated in the system to create a 4D-imaging capacity. The essential software to control both the stage and the shutter was written in JAVA and integrated in Lasersharp 2000 v5.2 software (BioRad, Hercules, CA). Images (800 × 600 pixels; 468 kb) of developing embryos were taken every 15 or 30 min in 20 different focal planes during the first 2 weeks and every 12 h during the remaining period using a CoolSNAP HQ CCD camera (Photometrics, Tucson, AZ). To create time-lapse videos the optical section of interest for each time point was manually selected. Subsequent imaging procedures were performed using ImageJ version 1.371 (available via http://rsb.info.nih.gov/ij/). Twenty embryos were monitored for viability after finishing recordings and all hatched in to J2 nematodes.

A total of 242 embryos were imaged to generate 242 videos of various lengths. The longest videos, which were derived from 6 embryos followed for 12.5 days, resulted in over 500 time points (frames). We followed about 20 embryos after imaging and they all hatch, which suggest that the treatment during imaging does not affect their development.

### Early cell lineage analysis

The early lineage was reconstructed using the Simi Biocell software (Simi Gmbh, D-85705 Unterschleissheim, Germany) based on three embryos, each developed up to the final embryonic stage [17]. Specific events were studied in additional embryos (*n* = 242) for confirmation. The terminology introduced by [18] for *C. elegans* development was followed to denote the different cells and stages during embryonic development. Herein “a” and “p” stand for anterior and posterior respectively, and “l” and “r” stand for left and right respectively.

### Immunohistochemistry

Western blotting was used to test the ability of the following three primary antibodies (obtained from the Hybridoma Bank at the University of Iowa) to cross-react with *M. incognita* proteins: *Tetrahymena* anti-α-tubulin (12G10; [19]), *C. elegans* anti-P-granule (K76, and OICD14; [20, 21]), and *C. elegans* anti-Par-3 (P4A1; [22]). Only the *Tetrahymena* anti-α-tubulin antibody showed cross reactivity (data not shown) and was used to conduct the immunohistochemistry experiments described here; the *C. elegans* antibodies tested did not react with *M. incognita* proteins.

Standard protocols developed by Susan Strome (University of California, Santa Cruz) were adapted for use with the nematode embryos. For each slide, freshly extracted *M. incognita* embryos were diluted to a concentration of 50 embryos/μl, and placed on a polylysine slide. A coverslip was placed on top of the embryos, and the slide was immediately transferred to dry ice. The coverslip was pressed down during the incubation on dry ice to ensure that the embryos adhered to the slide. The slide remained on dry ice until the embryos froze, after which the coverslip was quickly removed with a razor blade and the slide transferred to a coplin jar containing 100 % methanol at -20 °C, incubated for 10–15 min, then immediately transferred to a second coplin jar containing acetone at -20 °C, incubated for an additional 10–15 min. The slide was subsequently washed three times for, 5 min each, in a coplin jar containing freshly prepared phosphate buffered saline (PBS) with 0.1 % Tween-20, at room temperature. The slide was allowed to dry completely, and a perimeter denoted with a hydrophobic pen (Immedge™, Vector Laboratories, Inc., Burlingame, CA) around the region containing the embryos. The slide was incubated overnight at 4 °C with primary antibodies diluted 1:10 in PBS with 0.1 % Tween-20 and 0.1 % bovine serum albumin (BSA). The following day, the slide was washed with PBS with 0.1 % Tween-20 three times for five min each, and then incubated with FITC-conjugated anti-mouse secondary antibody, diluted 1:100 in PBS with 0.1 % Tween-20 and 0.1 % BSA, for two h at room temperature. The slide was again washed three times, 5 min each, with PBS with 0.1 % Tween-20 and a propidium iodide (PI) solution (0.2 μg/ml in PBS, 70 % glycerol) was added to the embryos and sealed with a coverslip and petroleum jelly or clear nail polish. These embryo containing slides were viewed and documented with an Olympus FluoView 300 confocal microscope.

## Results

### Egg characteristics

**Fig. 1:**
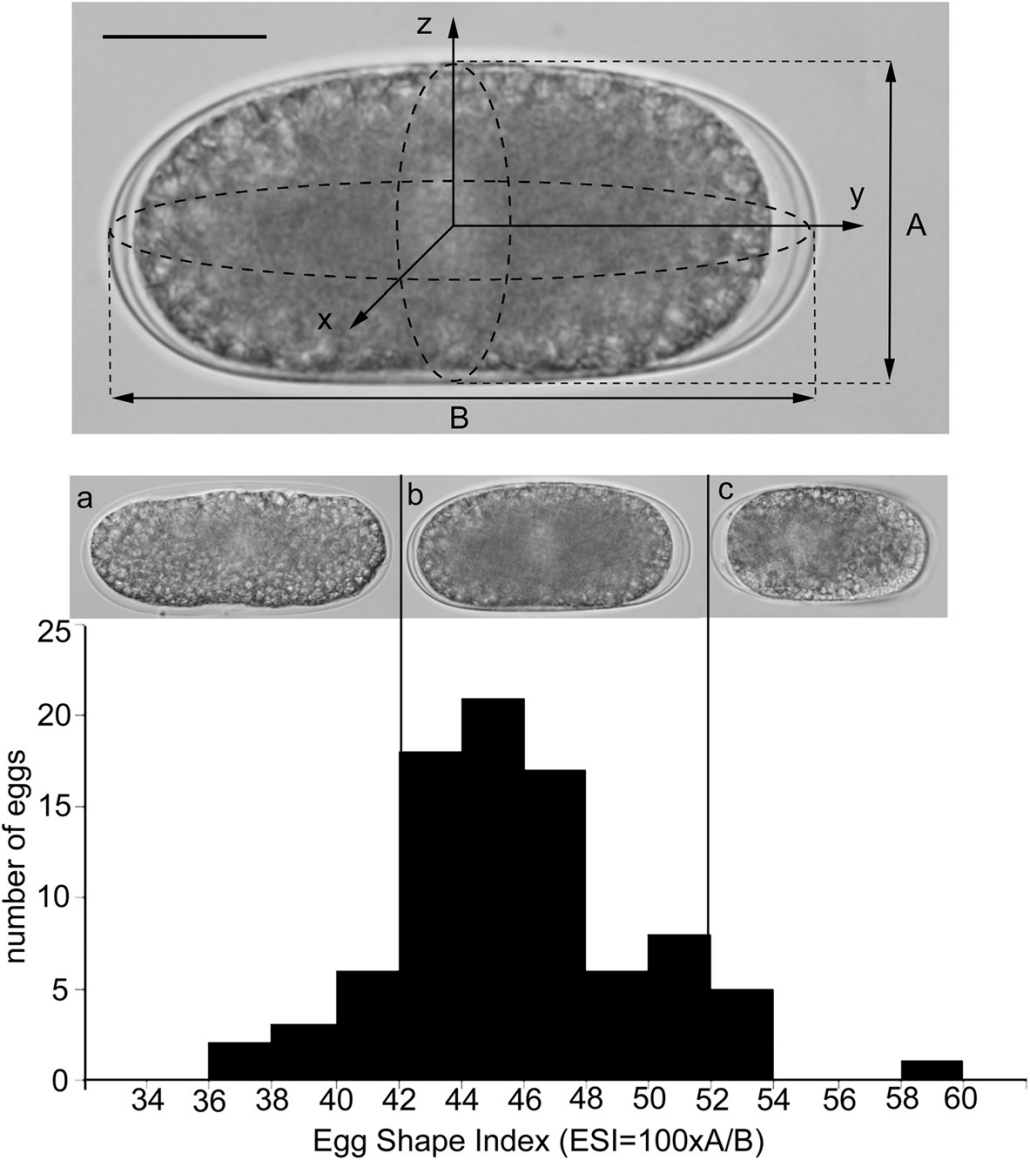
Shape of *Meloidogyne incognita* eggs. Distribution of the Egg Shape Index (ESI = 100 × A/B) calculated for 86 eggs of the root-knot nematode *M. incognita*. (**a**) elongated shape; (**b**) common ellipsoid shape; (**c**) stunted shape. Orientation: anterior, left. Bar = 25 μm

The average size of a viable egg was 94.37 μm (±5.71 μm) in length and 41.24 μm (±2.83 μm) in width (*n* = 86). These dimensions are comparable to the dimensions described for eggs of many nematode species [23]. However, our measurements revealed considerable intra-specific variation in the egg shape. Although the majority of the eggs were ellipsoid some were noticeably stunted while others were elongated. The roundness of each egg was defined by the Egg Shape Index (ESI), whereby the length of the shortest axis is divided by the length of the longest axis and multiplied by 100. The average ESI was 43.8 (±3.9) and the distribution of this parameter is shown in Fig. 1.

During maturation of the egg in the female, the cellular mass filled the egg completely. Prior to the initiation of embryonic development, the cell condenses and the lipoid membrane pulls away from the eggshell, leaving a perivitelline space at both poles of the egg. Centrally located in the granular cytoplasm, the nucleus was observable as a large distinct sphere. The developmental stage of the egg when deposited in the egg mass was the single- or two-cell embryonic stage. Only in rare cases further developmental stages could be detected in the reproductive system of older females. These may be the last eggs that are not laid by an exhausted female [1].

**Fig. 2:**
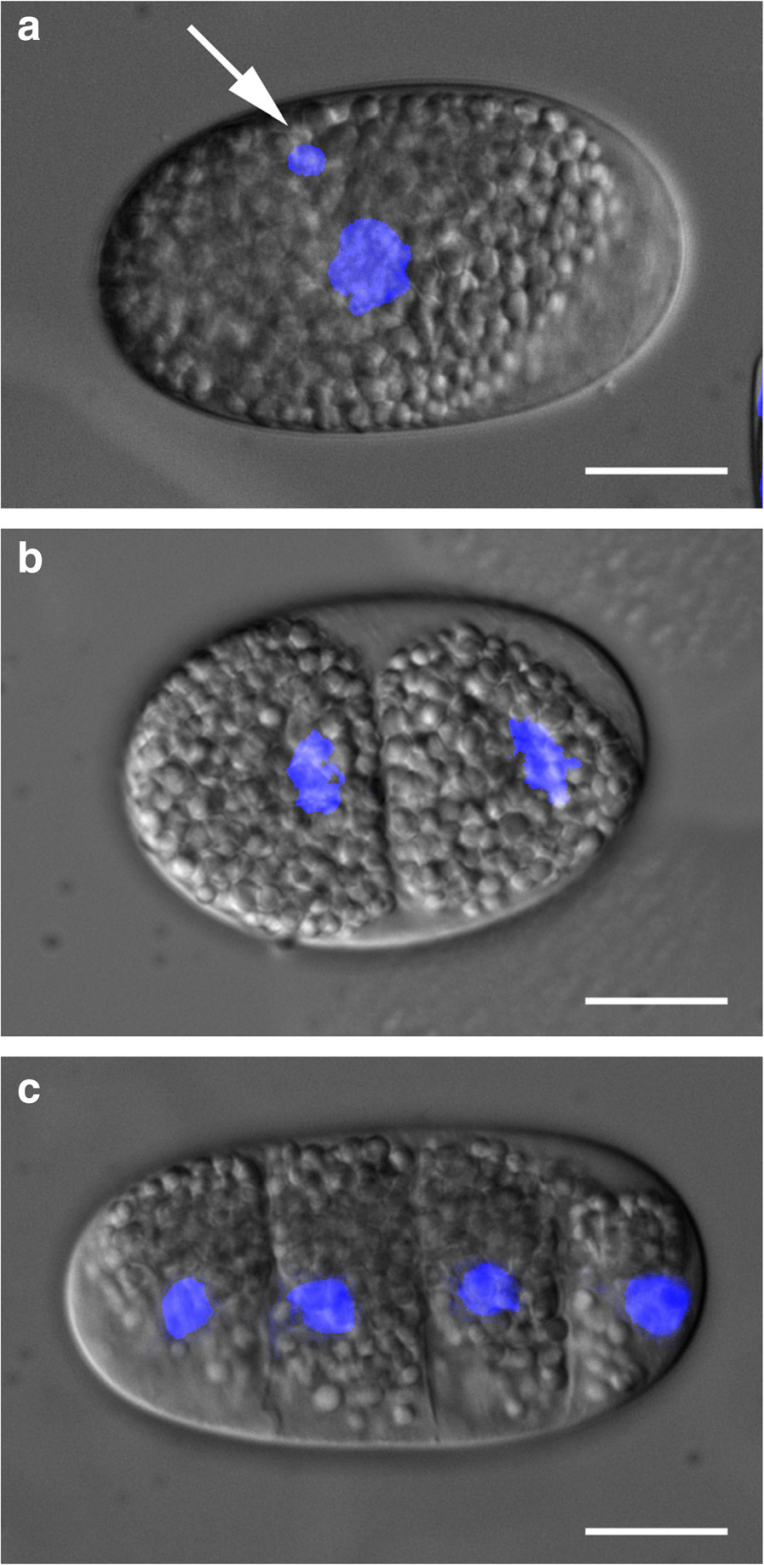
DNA staining of embryo during the first two cell divisions. Merged Nomarski and epifluorescence images to visualize DAPI-stained nuclei. **a** *M. incognita* uncleaved egg with one putative polar body in the cytoplasm (*arrow*). This putative polar body could not be detected in the later stages such as two-cell (**b**) and four-cell (**c**) stages. Bar = 25 μm

Since *M. incognita* reproduces by mitotic parthenogenesis, i.e. true asexual reproduction, in which no meiosis occurs but rather replication of the genome with subsequent production of a single polar body. [13, 24], one polar body was expected in the mature egg. However, this was never detected using light microscopy, likely resulting from the heavily granulated cytoplasm of the mature egg was which obscured subcellular details. A closer inspection of fixed and DAPI-stained eggs revealed a small DNA-containing cytoplasmic inclusion that may correspond to a polar body (Fig. 2a); similar bodies were observed when counter-staining with PI after immunohistochemistry (Fig. 7b, c and d). No defined polarity could be observed in the mature egg; therefore it was not possible to link the position of the inclusion to the posterior or anterior end of the egg. Similar bodies were seldom detected in later developmental stages, suggesting that they are rapidly degraded.

**Fig. 3:**
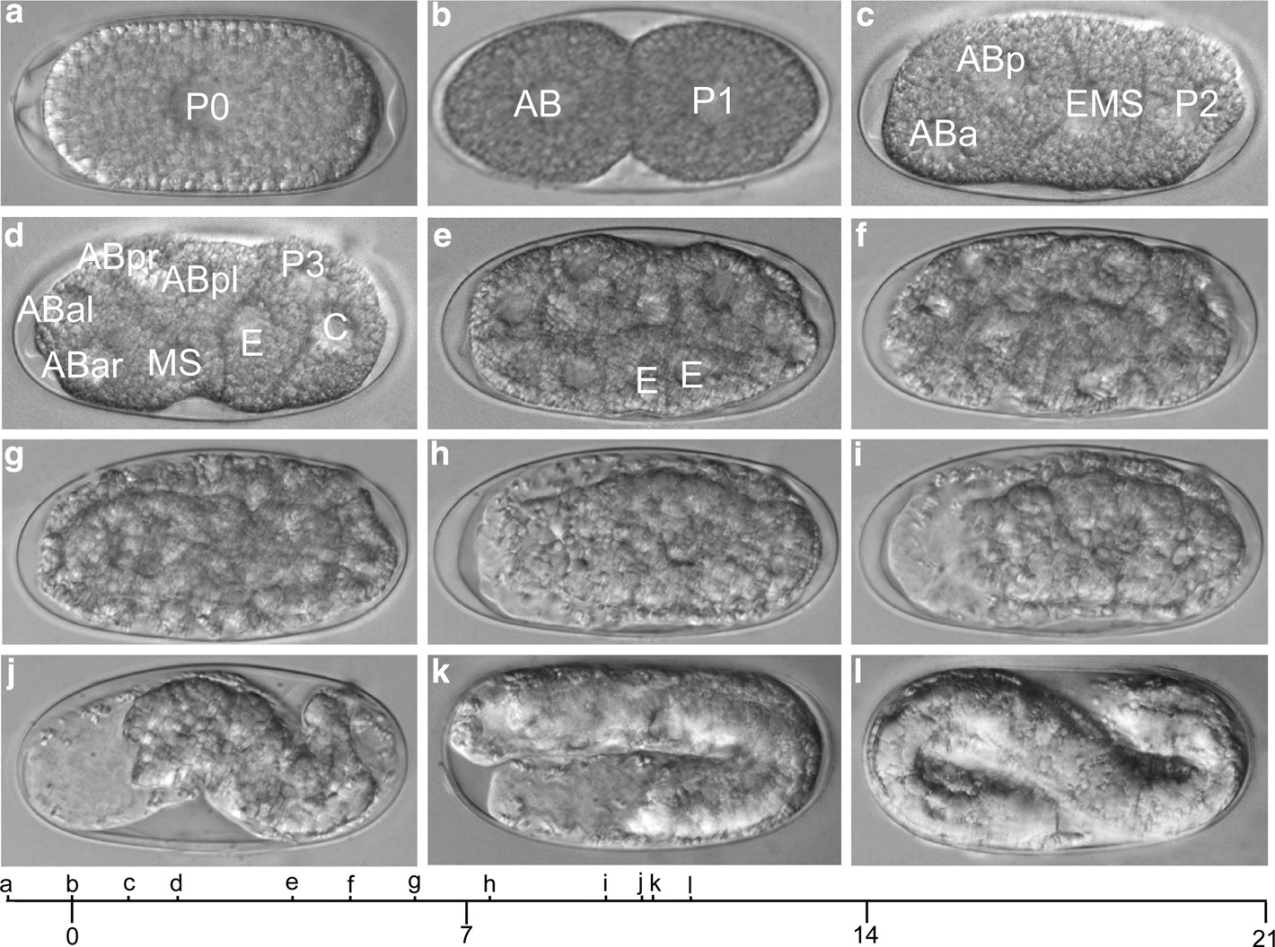
Embryos at different developmental stages. Characteristic stages in the embryonic development of *M. incognita* viewed by Nomarski DIC or bright field optics. A timescale in days after the first cell division is given at the bottom, and the corresponding pictures are indicated in this timescale. **a** single-cell stage; **b** 2-cell stage; **c** 4-cell stage with cells shifting from a linear towards a rhomboid pattern; **d** 8-cell stage prior to the division of the P_3_ cell; **e** embryo at the onset of gastrulation, both E-cells start to migrate inwards; **f** and **g** multiple cell stage showing clear differences between large endoderm cells surrounded by smaller ectoderm cells; **h** and **i** multiple cell stage showing clear difference between the light anterior pharynx part and a more dense granulated posterior part of the embryo; **j** elongation stage on the onset of bending in the eggshell; **k** 2-fold stage; **l** 3-fold (pretzel) stage. Orientation: anterior, left. Bar = 25 μm

**Fig. 4:**
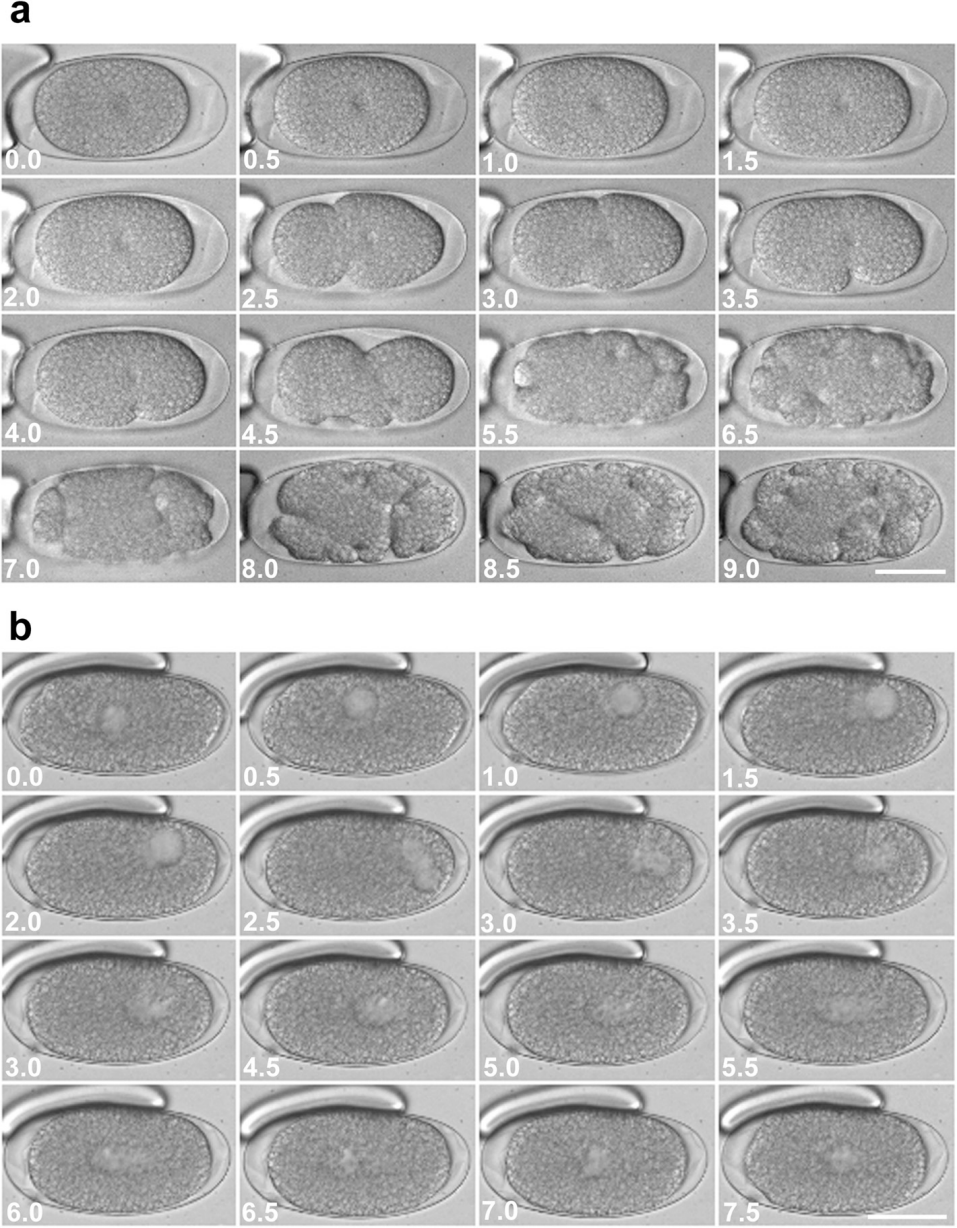
Pseudocleavages and nuclear migration prior to the first cell division. Selected images from digital time-lapse video recordings of living *M. incognita* embryos progressing through the early development. Images on (**a**) illustrate the pseudocleavages, high cytoplasmic activity and membrane ruffling that happen during the first cell cleavage. Images on (**b**) illustrate nuclear migration before the first cellular division. Both sets of images come from series in which images were taken every 30 min. The number in each picture is the time in hours after the first picture was taken. No cell divisions take place during either of these activities. Bar = 25 μm

**Fig. 5:**
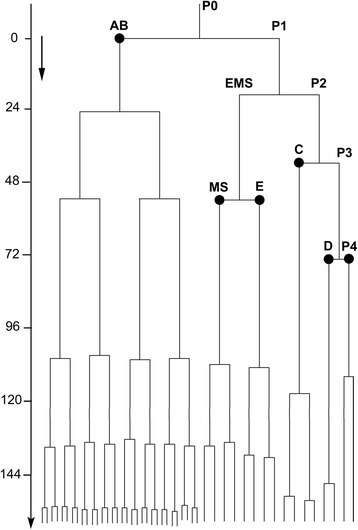
Early cell lineage of *M. incognita*. Cell lineage pattern of the early cleavages of the *M. incognita* embryo starting from the zygote to the 48-cell stage. The tree was built from data of a single representative specimen, and confirmed by two other specimens. The left scale indicates the hours after the first division. The formation of the six founder cells (the somatic cells AB, MS, E, C, D and the germline precursor cell P_4_ are marked in the lineage with a circle. The black arrow at the top left represents the time needed by *C. elegans* to develop from a fertilised egg to a hatched L1 stage at similar temperature

**Fig. 6:**
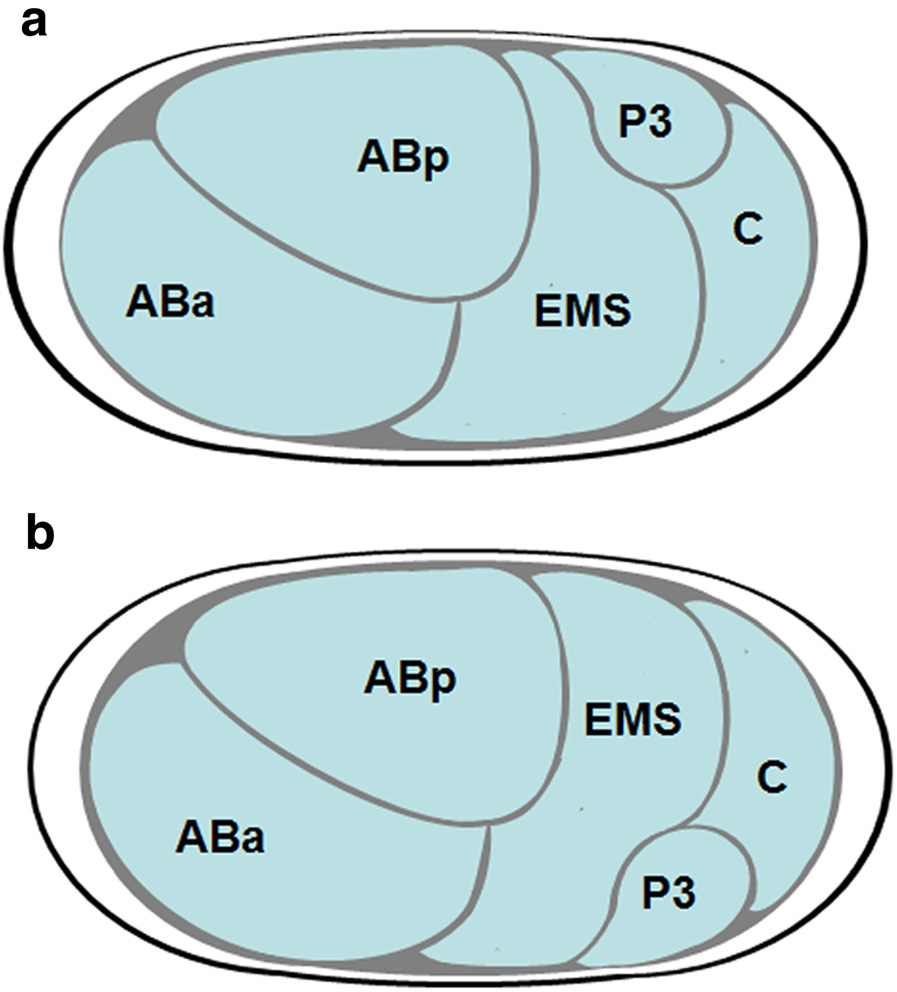
Spatial variation in the position of the P_3_ cell. After division of the P_2_ cell, the daughter P_3_ cell could be either in a ventral (**a**, *n* = 2) or dorsal position (**b**, *n* = 3)

**Fig. 7:**
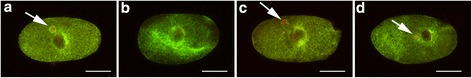
Cytoskeleton localization prior to the first cell division of *M. incognita* embryo. Four single-cell eggs after immunohistochemistry with anti-tubulin antibodies and counter stained with PI showing the localization of microtubules with respect to the nucleus and polar body (when visible). Microtubules can localize homogeneously throughout the cell (**a**), diagonally from one side of the cell to the other (**b**), on the opposite side of the polar body (**c**), or the same side of the polar body (**d**). White arrow indicates the position of the polar body. Bar = 25 μm

### Early cell lineage analysis

To study the early development of living embryos in detail, 4D-video recordings were made (Fig. 3; Additional file 1); in particular three recordings each representing the first 12.5 days of development were used to construct the early cell lineage. Prior to the first cell division, there was high cytoplasmic activity and extensive nuclear movement (Figs. 4 and 5). These events were accompanied with dramatic contractions and membrane ruffling of the single cell. During this period multiple pseudocleavage furrows could be seen, which distorted the shape of the cell (Fig. 4). The duration of this period was extremely variable, lasting less than one hour in some eggs to several days in others. Unfortunately, and since the exact moment when the eggs are laid is unknown, it is not possible to accurately determine if there is a pattern as to how long it takes for the eggs to enter the first cell division. Having said this, 50 eggs (at the one cell stage) were mounted and imaged, and the time between mounting and the first cell division was estimated. From this survey it appears that eggs can stay for long periods of time without undergoing development (up to 60 h). Once development started, all embryos took a similar time to complete early development, although none of these embryos was followed to determine if they would hatch to produce J2s.

Using 4D-microscopy, the early cell lineage of *M. incognita* was established, allowing the timing, location and ancestral relationship of each division during development to be known (Fig. 6). The early embryonic development of *M. incognita* showed a fixed cleavage pattern (summarized in Table 1). The first cell division was unequal, although the size difference between both daughter cells was not always obvious; a survey of 32 embryos after the first cell division indicated a ratio of 2:1 unequal cell divisions: equal cell divisions. The asymmetry defined the A-P axis of the embryo and the daughter cells formed were an anterior somatic blastomere (AB) and a slightly smaller posterior germline cell (P_1_) in case of an unequal division (see Additional file 2: Figure S1). The germline cell has the potential to produce a somatic cell and a new germline cell with each cleavage. The annotation of the germline cells in the early cell lineage was based on cell size, their position in the developing embryo, and their specific cleavage pattern. The germline cell P_1_ divided, forming the second somatic blastomere (EMS) and the germline cell P_2_. This division was followed by the cleavage of the blastomere AB. Since cleavage of AB occurred prior to further cleavages of the P-lineage, the development pattern is described as being synchronous [15], although the cleavages of AB and P_1_ do not occur simultaneously (the four blastomeres present are the same generation). At the four-cell stage the blastomeres present were ABa, ABp, EMS and P_2_ in a linear pattern (Fig. 2c). In the elongated eggs, the ABp blastomere migrated towards the future dorsal side of the embryo, ending in an arrangement resembling the oblique four-cell stage of *C. elegans*. In stunted eggs (ESI > 50) the linear pattern was never achieved. Instead, the four cells were immediately squeezed in the rhomboidal-like position, which may be caused by constraints imposed by the eggshell (see Additional file 3: Figure S2). This arrangement of cells was followed by the cleavage of the posterior P_2_ germline cell, producing the C founder cell and the germline cell P_3_. At this point we observed variations in spatial patterns. After the division of P_2_, P_3_ could either be in a ventral (50 % of the observed embryos) or dorsal (50 % of the observed embryos) position (Fig. 7). At the second division round of AB into a left pair and a right pair, the anterior daughters, ABal and ABpl, are skewed into the anterior direction. Because of this shift to the anterior, bilateral symmetry does not become obvious in early embryonic development. Due to heavy granulation of the cells, recordings could not be followed through complete development, so it was not possible to determine how bilateral symmetry in the juvenile body plan was achieved. The division of the AB cell was followed by the division of the EMS cell, resulting in the creation of two additional founder cells, MS and E. The last founder cells were formed by the next cell division, where the germline cell P_3_ produced the germline cell P_4_ and the somatic cell D. In embryos with P_3_ in a ventral position, the division of P_3_ resulted in a posterior D cell and an anterior P_4_ cell, leading to the configuration E-P_4_-D-C (from ventral to dorsal), as described for *C. elegans*. Alternatively, in embryos with P_3_ in a dorsal position the cleavage polarity of P_3_ was reversed, leading to the configuration E-C-D-P_4_. In *Meloidogyne* embryos, contact between germline and the endodermal progenitor was restored when C started to migrate more dorsally and P_4_ and D switched positions, until the configuration P_4_-D-C was achieved. All six founder cells were present 3 days after the first cleavage. The successors of each founder cell divided almost synchronously, resulting in a specific order of cleaving cell groups. In addition, specific cell-cell contacts, which specify the fates of the AB lineage *in C. elegans* [25–27], were observed in *M. incognita*: ABara and ABalp always contacted the MS cell, while their bilateral counterparts did not.

**Table 1:** Sequence of the first divisions

*C.elegans*		*M. incognita*	
Dividing cell	Cell number after division	Dividing cell	Cell number after division
**P**_**0**_	**2**	**P**_**0**_	**2**
1AB2	3	P_1_	3
P_1_	4	1AB2	4
2AB4	6	**P**_**2**_	**5**
**EMS**	**7**	2AB4	7
**P**_**2**_	**8**	**EMS**	**8**
4AB8	12	**P**_**3**_	9
1MS2	13	4AB8	13
1E2	14	1MS2	14
1C2	15	1E2	15
8AB16	23	P_4_	16
**P**_**3**_	**24**	1C2	17
2MS4	26	8AB16	25
2C4	28	2MS4	27
16AB32	44	2E4	29
2E4	46	1D2	30
1D2	47	2C4	32
4MS8	51	16AB32	48

Comparison of the early cell division sequence between *C. elegans* and *M. incognita*. Divisions that lead to a founder cell (AB, MS, E, C, D and P_4_) are in bold. The cell numbers after the division are given, demonstrating that P_4_ is present in the 24-cell stage in *C. elegans*, compared to the 9-cell stage in *M. incognita*

### Gastrulation

In *M. incognita* gastrulation started around 131 h, in the 26-cell stage (Fig. 3e). Like in *C. elegans*, the two daughter cells of the E cell were translocated from the ventral side to the center of the embryo. The posterior endodermal precursor cell Ep first migrated inwards between the four MS granddaughters MSaa, MSap, MSpa and MSpp and was then followed by its anterior sister cell, after which they divided left-right. Tracking individual cells beyond this stage (48 cells) was difficult because heavy granulation of the cells obscured details. As a result, cell divisions were not followed in detail beyond this time point, but some specific developmental stages could be distinguished.

Cell differentiation after gastrulation resulted in the formation of large endodermal cells surrounded by smaller ectodermal cells (Fig. 3g). Both layers developed further and resulted in the formation of two zones of dissimilar density within the embryo, a light anterior area packed with cells (as demonstrated with DAPI staining; data not shown) and a dark posterior zone (Fig. 3i). After the spheroid embryo started to elongate, it was squeezed against the eggshell and forced to bend, forming a worm-like two-fold embryo (Fig. 3j-k). Compared to the total early developmental time (approximately 21 days), elongation of the embryo took place quite rapidly (approximately 3 days) and resulted in a first stage juvenile, coiled three to four times within the eggshell. As soon as the nematode started to elongate, it moved frequently and vigorously, indicating that body muscles had started to function. These movements continued until the hatching of the J2 approximately 21 days after the first cell division.

### Cytoskeleton organization at the single cell stage and during early cell divisions

**Fig. 8:**
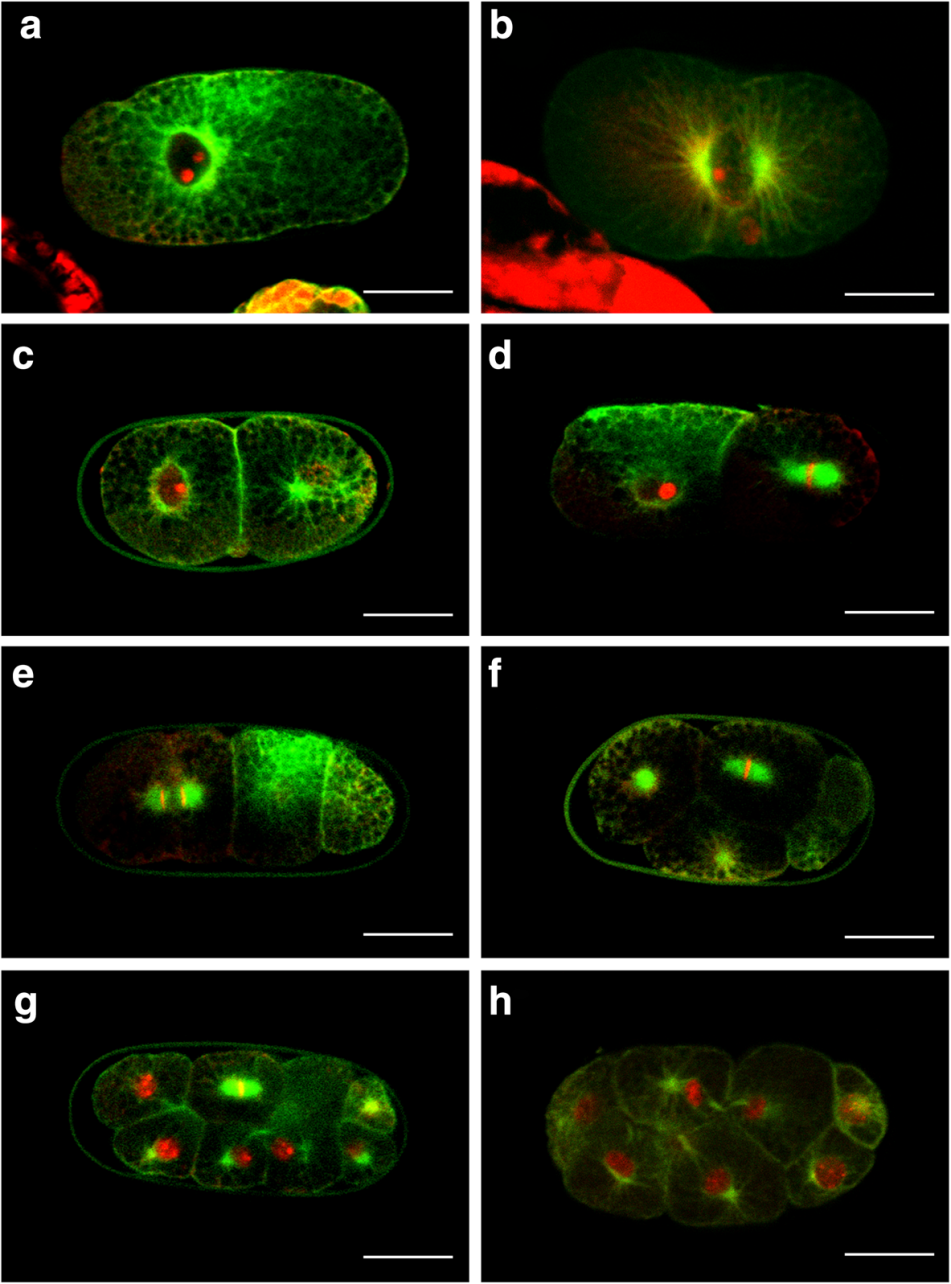
Cytoskeleton localization during early cell divisions of *M. incognita* embryos. Collage of single cell egg and embryos illustrating the different cell divisions of early development in *M. incognita*. **a** Single cell egg; **b** Single cell activated to continue with development and at the initial stages of cell division. The two MTOC are clearly visible and are parallel to the A-P axis; **c** Two cell embryo; the posterior cell shows the two MTOC. A polar body is still visible at the bottom between the two cells; **d** Two cell embryo with the posterior cell at metaphase of the second cell division; **e** three cell stage with the anterior cell at anaphase of the second cell division; **f** Six cell stage (the P_2_ has already divided to generate C and P_3_) with ABa, ABp and EMS in a triangle. EMS cell is at metaphase of the cell division; **g** eight cell stage; **h** advanced eight cell stage. Orientation: anterior is left (on images from **c** to **h**) and dorsal is top (on images from **f** to **h**). Bar = 25 μm

Since *M. incognita* has a mitotic parthenogenetic mode of reproduction we wanted to study the distribution of microtubules during the single cell stage and within the first cell divisions of the embryo, in order to compare them to sexually reproducing nematodes such as *C. elegans* (free living) and *Bursaphelenchus xylophilus* (plant parasitic). One single polar body was visible in most eggs, although its localization varied from the cell periphery to close proximity to the nucleus, and the nucleus was seen at various locations in the cell. Distribution of microtubules in single celled eggs varied from homogeneously distributed strands in the cytoplasm (Fig. 7a) to concentrated strands going diagonally from one side of the egg to the other (Fig. 7b). In other eggs microtubule strands concentrated on one side of the egg and distributed more homogenously (Figs. 7c and d, and 8a). In addition, microtubules were concentrated around the nucleus but preferentially towards only one side; generally the amount of microtubules around the nucleus was greatest opposite the visible polar body (Figs. 7). In some eggs the microtubules strands around the nucleus were concentrated in two opposing sites perhaps serving as Microtubule Organizing Centers (MTOC).

**Fig. 9:**
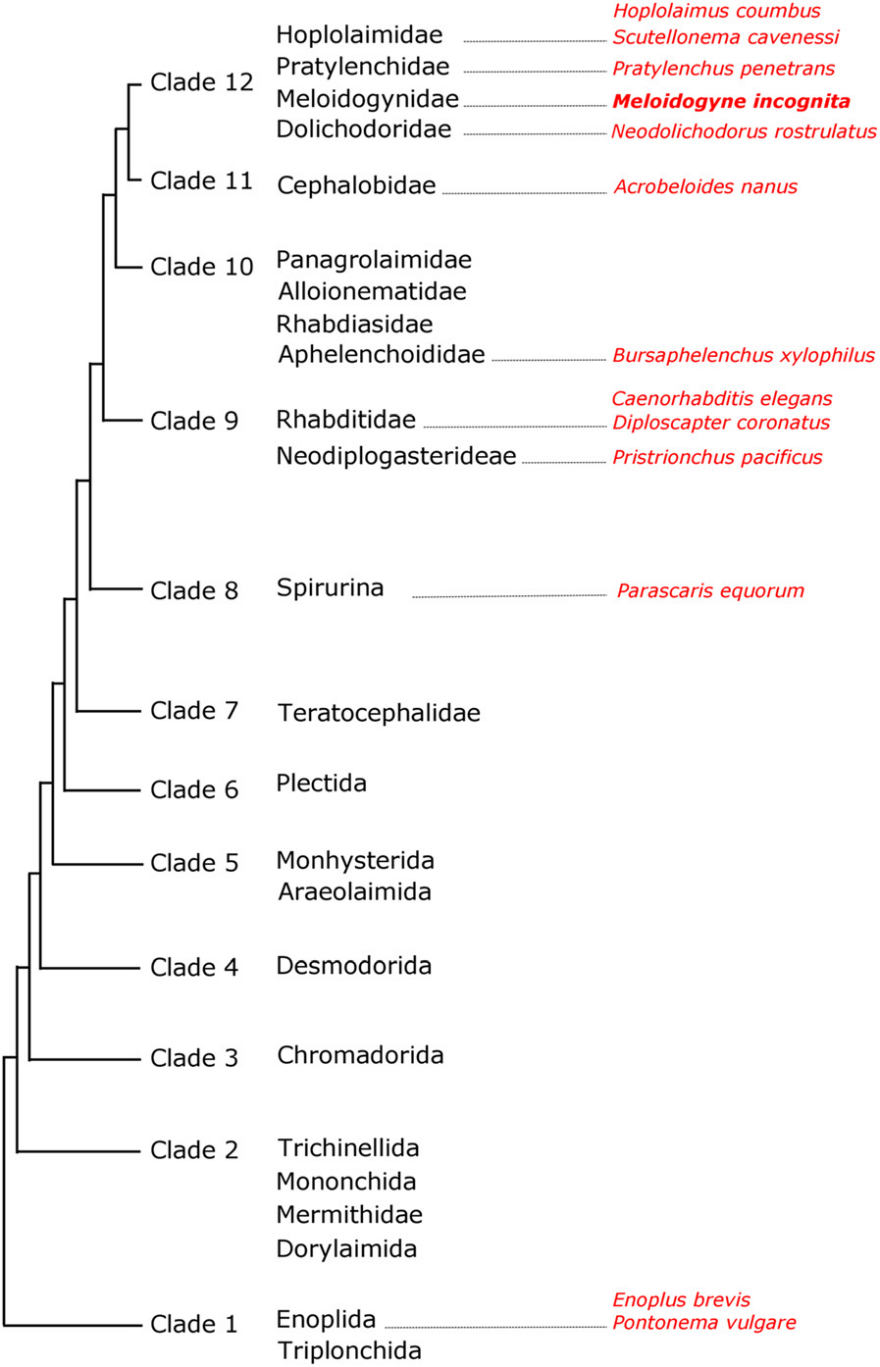
Phylogenetic tree for the phylum Nematoda. A summarized version of a phylogenetic tree based on 18SrDNA developed by Holterman et al. [47]. Twelve major clades are identified as well as the species listed in the text

Microtubules organization in opposite sides of the nucleus reached a maximum at the time when a line going through the two MTOC was parallel to the longer axis of the cell (Fig. 8b). After the first cell division (as seen in most of the studied lineages) the A-P axis was determined. In those eggs where the division was unequal, the smaller cell was the posterior (P_1_; posterior lineage) cell and the larger was the anterior (AB) cell (Fig. 8c). In the posterior cell, and prior to the next cell division, the two MTOC were localized along the A-P axis and the cell’s DNA could be clearly seen between them (Fig. 8d; in this image the P_1_ cell is at metaphase). Similarly, and prior to the cell division of the anterior AB cell, the MTOCs were localized parallel to the A-P axis (Fig. 8e; in this image the AB cell is at anaphase). After generation of the four blastomere stages (ABa, Abp, EMS and P_2_), the cells adjusted their positions so that the ABa and EMS cells were touching each other on what will become the ventral surface of the embryo, and the P_2_ cell divided to generate C and P_3_; at this stage EMS began to divide and the MTOCs organized parallel to the A-P axis (Fig. 8f).

## Discussion

### *M. incognita* eggs are characterized by high cytoplasmic activity and extensive nuclear movement

The ellipsoid eggs of the root-knot nematode, *M. incognita*, lack overall uniformity. This observation has been recorded for the eggs of several nematodes [28, 29] and it was even suggested that intra-specific differences might be as great as inter-specific variations (described in [23]). Individual eggs were followed by 4D-microscopy during the developmental process to study successive cell divisions.

Two events that show similarity between *C. elegans* and *M. incognita*, prior to the first cell division, are cytoplasmic ruffling and nuclear migration. In *C. elegans,* adjustments are made to the newly fertilized embryo; cortical proteins, other cytoplasmic material and organelles are moved to defined regions by the cytoskeleton. These movements are reflected morphologically as a series of cytoplasmic ingressions and invaginations in the cell membrane [11, 30]. Proteins and ribonucleoproteins, such as the PAR proteins and P-granules, respectively, are repositioned in the newly fertilized zygote [31]. Similarly, cytoplasmic ruffling takes place in the single-celled embryo of *M. incognita*, suggesting that there is a need to organize the embryo prior to the first cell division. However, it is unclear whether the redistribution of cytoplasmic materials and/or the unequal size of the daughter cells are responsible for determination of the A-P axis. It is also unclear whether a PAR-3-like mechanism is involved, since the antibodies tested did not return cross-reactivity. Further testing is required to demonstrate whether or not a PAR-3 like mechanism exists, and whether it is involved in the development of *M. incognita*. It is also possible that other PAR proteins, such as PAR-1, −2, −4 or −6, are involved; each of these proteins are known to be evolutionarily conserved in many organisms and function in cell polarization during development [32].

Multiple pathways initiate embryogenesis across many different species of nematodes. For instance, electrical pulses, changes in calcium levels or physical disruption of the oocyte’s membrane (poking) can initiate embryogenesis in some species [33, 34]. In *C. elegans*, an enucleated sperm was shown to initiate embryogenesis, activating the oocyte and leading to the first cell division [34]. Few studies have described the embryonic development in parthenogenetic nematode species, and the nature of the signals that trigger embryonic development are not yet clear. Lahl et al. [35] describe the development of three free living parthenogenetic nematodes: *Acrobeloides nanus, Diploscapter coronatus* and *Plectus* sp. In these species, the eggs were deposited as single-celled embryos into the environment, and there was no evidence for oocyte organization prior to the first cell division. Furthermore, the single-celled eggs of *M. incognita* initially have a soft chorion before they are deposited into the external environment; only then do the chorionic shells harden. The vulva of the female compresses the egg when it is deposited into the gel matrix (female extracellular secretions, which will hold the eggs/embryos) and it is possible that this physical action is the trigger to initiate development. In our study with *M. incognita*, we only observed single-celled embryos transferred through the reproductive tract of the female, which are deposited as single- or two celled embryos into the egg mass. The eggs seem to be dormant for long, variable intervals (in one recording the egg was dormant for 4.5 days; see Additional file 4), and then a currently unidentified trigger initiates embryogenesis. As in other animals, it is possible that nuclear movement defines where the cleavage furrow will form, and whether the first cell division will be asymmetrical [36]. Nuclear positioning in *C. elegans* is a PAR-3-dependent process, but it is unclear whether a similar mechanism is involved in *M. incognita.*

We identified the presence of polymerized α-tubulin in developing *M. incognita* embryos, using antibodies that have been shown to react with the α-tubulin of several diverse species [19]. Using time-lapse microscopy and immunohistochemistry, we observed both cytosol containing a high density of smaller particles and a tightly knit microtubules in one highly compacted region of the single-celled embryo. Prior to the first cell division, the nucleus migrated away from the high density, small-granule region into the low density large-granule region, where the first asymmetrical cell division took place. It is possible that the combination of high cytoplasmic activity, extensive nuclear movement and the contractions and membrane ruffling of the single cell are caused by the constant reorganization of components of the cytoskeleton, for example contractility of the actin cytoskeleton as it is the case in *C. elegans* [7, 37]. Since the polarity cue in *M. incognita* is unknown (because no sperm contribution), it is tempting to speculate a self-activation mechanism of the actin cytoskeleton. The first cell division, generating the AB and P_1_ cell, are always located in the low-density region of the single cell. Hence, it is tempting to speculate that the cytoplasmic density gradient and asymmetrical distribution of the cytoskeleton induced a first asymmetric division. Our results of cytoplasmic rearrangements prior to the first cell division in *M. incognita* seem to contradict earlier findings [38], and we have no explanation for this contradiction.

### *M. incognita* has a slow developmental tempo

*M. incognita* showed an extremely slow embryonic development compared to *C. elegans*; the development was at least 35 times slower (development from first cell cleavage to hatching almost 500 h in *M. incognita* and 14 h in *C. elegans* at room temperature). For early embryogenic events this difference is even grater: the time between division of the P_0_ and the division of the somatic founder cell E is less than 1 h for *C. elegans* (47 min), but approximately 114 h for *M. incognita*. Similarly, the completion of a cell cycle during early embryogenesis required several hours, whereas in early developmental stages *C. elegans* cells divide approximately every 10 min.

The optimal temperature for development of *M. incognita* is 28 °C, and corresponds to the geographical distribution of this nematode in subtropical regions. At this temperature it takes approximately 3 weeks to complete one life cycle. Higher temperatures (above 30 °C) have a devastating effect on the survival rate and lower temperatures result in an extension of the life cycle (discussed in Tyler, 1933b [39]). However, the high optimal temperature (28 °C) had a disturbing effect on the agar pad and resulted in out of focus drifts during imaging; this drift could be a consequence of the objective lens contacting the cover slip and the continuous moving of the slide from embryo to embryo causing a change in pressure and resulting in the out of focus drift. For this reason we chose to follow the development at room temperature (22 °C ± 1 °C). The use of this suboptimal temperature could partly explain the slow embryonic development, but it is unlikely that it affected the sequence of cell divisions [40, 41]. Vangestel et al. [42] studied the influence of temperature (15 °C, 20 °C and 25 °C) on early embryonic development in *Pristionchus pacificus* (Neodiplogasteridae) and found that besides developmental tempo, all the other examined parameters were found to be similar at different temperatures (division sequence, time of establishment of the P_4_ cell, gastrulation and cell-cell contacts). The slow development of *M. incognita* appears to be typical for tylenchids since similar times have been described for *Neodolichodorus rostrulatus* [9–10 days; [43]], *Scutellonema cavenessi* [10–11 days; [44]], *Pratylenchus penetrans* and *P. zeae* [10 days; [45]], and *Hoplolaimus columbus* [12 days; [46]]. Slowly developing nematodes are also found in clade 1 (Fig. 9) of the phylogeny of Holterman et al. [47]. In the marine nematodes *Enoplus brevis* and *Pontonema vulgare* the time until hatching is 16–20 days at 25 °C [48] and 30 days at 16 °C [49], respectively. These enoplids of clade 1 (clades as defined in [47]) are found in a stable marine habitat. Schierenberg formulated that this slow development was probably necessary to preserve aspects of regulative development. Associated with the colonization of freshwater and terrestrial habitats, nematodes possibly needed to respond to more rapidly changing environments and thus nematodes that developed faster, or nematodes that were more tolerant to changing environmental conditions, had a selective advantage [50]. Besides these slowly developing nematodes from clade 1 and *M. incognita* in clade 12, which shows a very slow development, more slowly developing nematodes were also found in other clades, e.g. *Parascaris equorum* (clade 8) having an embryonic development of 1–2 weeks at room temperature (28 °C). Hence, it appears that the speed of development has changed independently in several taxa.

### The embryonic development shows similarities to *C. elegans*, but also features typical for Cephalobomorpha

*M. incognita* has a fixed cleavage pattern and the development is described as synchronous [15], similar to *C. elegans*. The cell-cell contacts MS-ABara and MS-alp, which induce pharyngeal potential in those cells in the *C. elegans* embryo [25–27], were also identified in *M. incognita*. As in *C. elegans*, only one member of two bilateral homologs of blastomeres came into contact with the signaling blastomere MS. Whether signaling from MS effectively results in left-right asymmetries through the Notch pathway, as in *C. elegans*, remains to be determined (reviewed by [51].

Essential differences from *C. elegans* exist in the timing of germline divisions. While in *C. elegans* the primordial germ cell P_4_ is present in the 24 cell stage, P_4_ is already present in the 9 cell stage in *M. incognita.* This is in contrast to the previous findings of Dolinski et al. [15], who reported a simultaneous cleaving for the EMS and P_3_ cell. Cephalobidae, however, are characterized by the division sequence P_1_-P_2_-AB-P_3_. Here, the P_4_ cell is established already at the 6-cell stage. Skiba and Schierenberg [52] stated that in these slow developing embryos the germline is separated relatively early, to preserve germline quality. Examining the configuration of the posterior cells, we observed a variable pattern. In *C. elegans* the germline daughter cells P_0_ and P_1_ are always positioned posterior to their somatic sister cell, while after the division of P_2_, the germline cell P_3_ will be in an anterior position and its somatic sister C in a posterior position. This phenomenon is described as “reversal of cleavage polarity” [PR; [53]]. In *M. incognita*, the position of the germline cell P_3_ was not determined and can be both posterior and anterior to its somatic C cell. This variable configuration is in contrast to observations of Goldstein et al., [3] who stated an absence of polarity reversal for *M. incognita*. The variations in spatial patterns are not unique to *M. incognita*, but were first described for *Cephalobus* sp. (later referred to as *Acobeloides nanus*) by Skiba & Schierenberg [52]. In this species the alternative orientation of the cleavage spindle in AB results in 2 different arrangements of blastomeres in the 5-cell stage [50, 52]. In *M. incognita* however, this did not depend on the orientation of AB’s cleavage spindle as the two AB cells were already present when P_2_ divided. This variable spatial pattern was also observed in four other members of the Cephalobomorpha, and is possibly a synapomorphy for Tylenchomorpha and Cephalobomorpha [42]. Nevertheless, the variable configuration of these cells is also found in other clades. Using experimental interference, Laugsch and Schierenberg [28] found variable configurations in three *Rhabditis* species and Lahl et al. [54] mentioned variable configurations for *Diploscapter coronatus*. In these nematode embryos, subsequent cellular migrations restore contact between the germline and the endodermal precursors, leading to the *C. elegans* spatial arrangement (P_4_-D-C) before the onset of gastrulation. Skiba and Schierenberg [52] suggested that this configuration is required for further normal development. This phenomenon is also observed in other species: in *Drosophila*, *Xenopus*, chick and mouse the primordial germ cells associate with the developing gut, from which they migrate to the gonads during organogenesis [55].

## Conclusions

The work presented here on the early development of the apomictic *M. incognita* reveals some common features with the early development of the sexually reproducing *C. elegans* such as cytoplasmic rearrangements prior to the first cell division, a fixed cleavage pattern and early synchronous development. However, major differences also exist such as the timing of germline divisions (P_4_ being present in the 9-cell stage), the developmental tempo (at least 35 times slower in *C. elegans*), and the variable configuration of the posterior cells.

### Ethics approval and consent to participate

Ethics was not required for this study.

### Consent for publication

Not applicable.

### Availability of data and material

The data supporting the conclusions of this article is included within the article and its additional file(s).

## Additional files

Additional file 1: Time-lapse video 1. This is a time-lapse video of a *Meloidogyne incognita* embryo during the first 12.5 days (529 frames at 30 min intervals) of development. The future anterior (a), posterior (p), dorsal (d), and ventral (v) sides of the embryo are indicated. (MP4 7327 kb) Additional file 2: Figure S1. Level of asymmetry between AB and P_1_ blastomeres in *M. incognita*. To illustrate the level of asymmetry found in approximately 50 % of the embryos observed, six such embryos are shown here. An estimation of the ratio of the area of AB (cell on the left side of each image) versus P_1_ for these six embryos gives a mean value of 1.32 with a standard deviation of 0.22. Cell areas were estimated using the area function of ImageJ and the AB cell value was divided by the P_1_ cell value. Bar = 25 μm. (TIF 5146 kb) Additional file 3: Figure S2. Rhomboidal-like cell positioning in stunted eggs. The images (a, b, c and d) are from the same embryo at the two cell stage (a) and going into the four cell stage where the rhomboidal-like cell positioning is visible. Bar = 25 μm. (TIF 1869 kb) Additional file 4: Time-lapse video 2. This is a two part time-lapse video: the first part is of a *Meloidogyne incognita* embryo prior to the first cell division and illustrating pseudocleavage activity during 4.5 days (225 frames at 30 min intervals), and the second part is of a *Meloidogyne incognita* embryo prior to the first cell division and illustrating nuclear movement during 8 h (16 frames at 30 min intervals). (MP4 5189 kb)
