# Supplementary figures and images for: Early development of the root-knot nematode Meloidogyne incognita
Source: BMC Dev Biol. 2016 Apr 28;16:10. doi: 10.1186/s12861-016-0109-x (PMC4848817; doi:10.1186/s12861-016-0109-x)

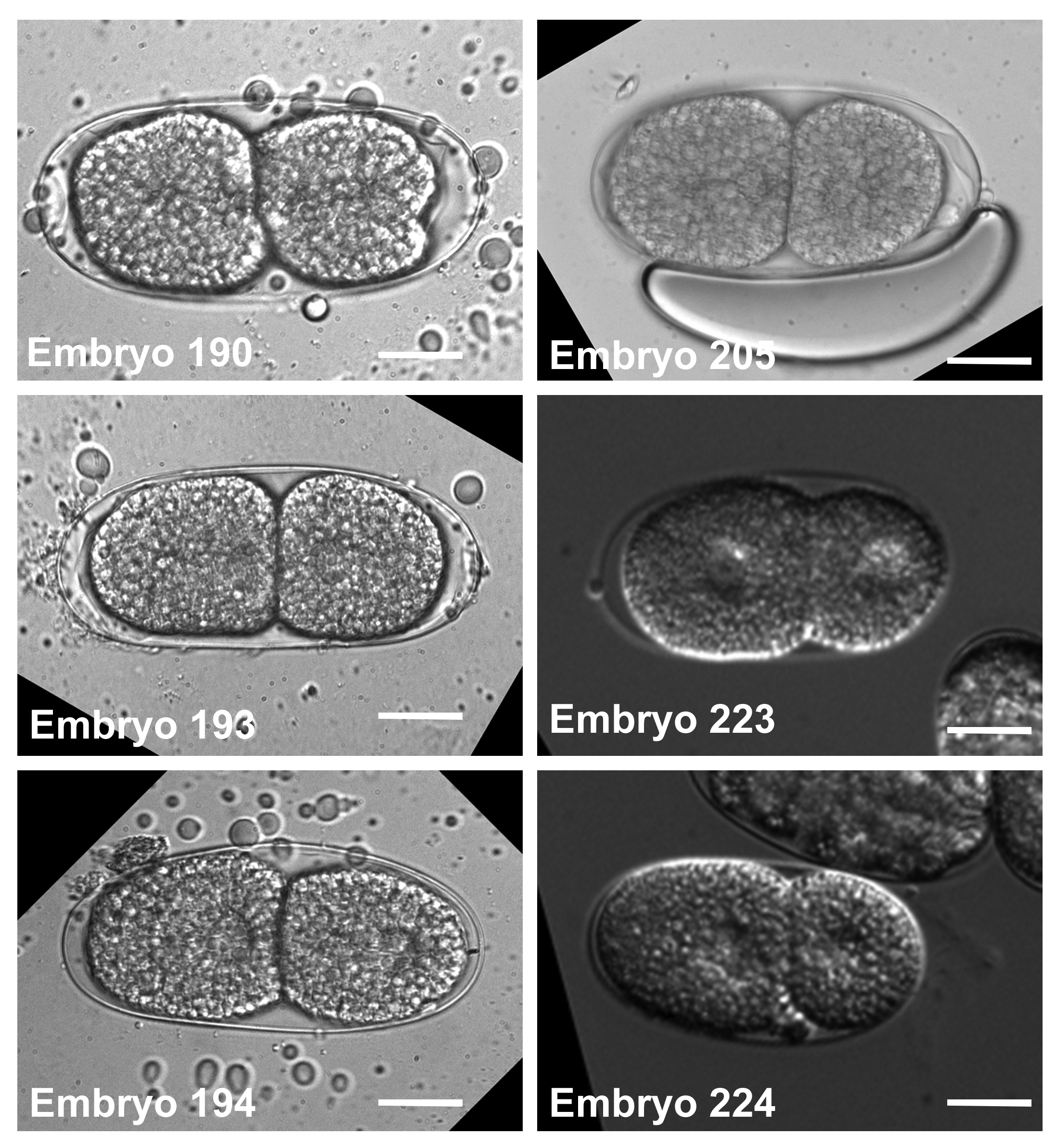

Supplement: Additional file 2: Figure S1. — Level of asymmetry between AB and P1 blastomeres in M. incognita. To illustrate the level of asymmetry found in approximately 50 % of the embryos observed, six such embryos are shown here. An estimation of the ratio of the area of AB (cell on the left side of each image) versus P1 for these six embryos gives a mean value of 1.32 with a standard deviation of 0.22. Cell areas were estimated using the area function of ImageJ and the AB cell value was divided by the P1 cell value. Bar = 25 μm. (TIF 5146 kb) [file 12861_2016_109_MOESM2_ESM.tif]

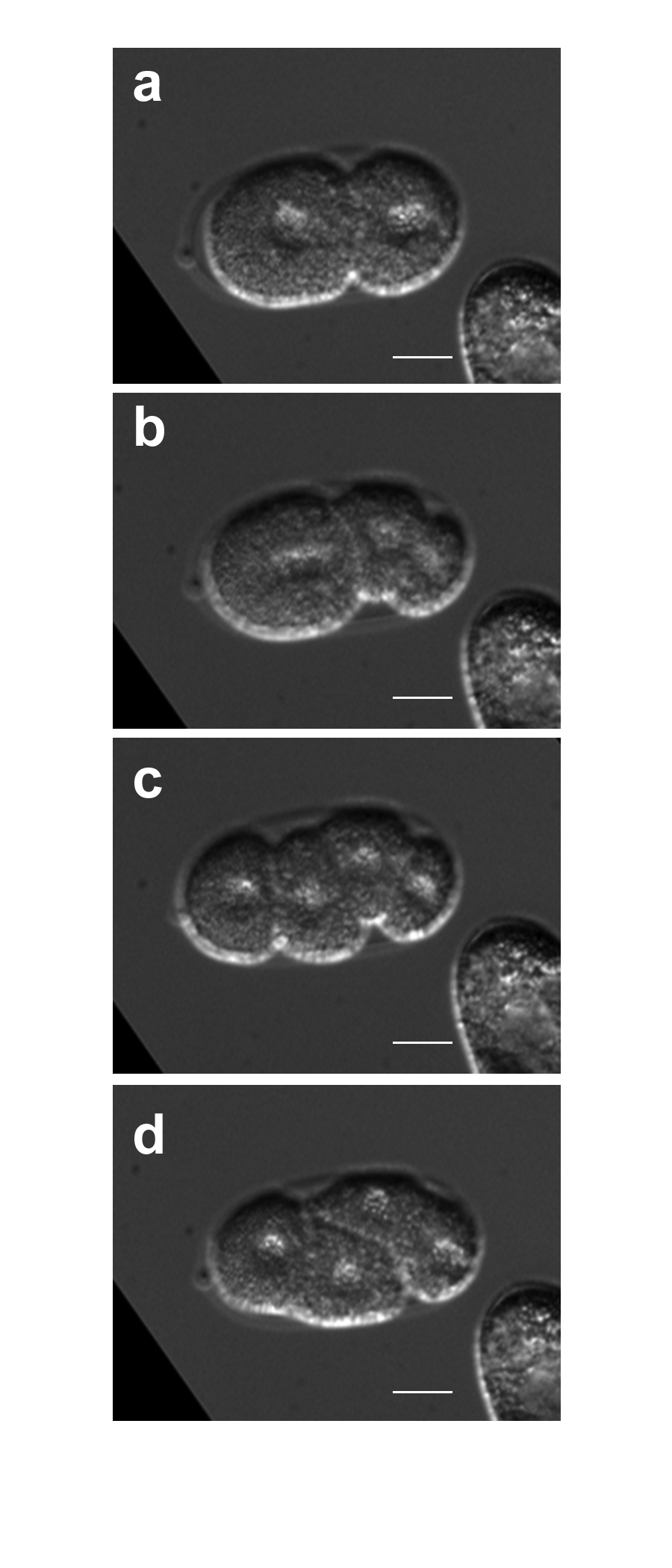

Supplement: Additional file 3: Figure S2. — Rhomboidal-like cell positioning in stunted eggs. The images (a, b, c and d) are from the same embryo at the two cell stage (a) and going into the four cell stage where the rhomboidal-like cell positioning is visible. Bar = 25 μm. (TIF 1869 kb) [file 12861_2016_109_MOESM3_ESM.tif]
